# The genome sequence of the Blind Ringlet, *Erebia pharte* (Hubner, 1803-1804) (Lepidoptera: Nymphalidae)

**DOI:** 10.12688/wellcomeopenres.27705.1

**Published:** 2026-09-04

**Authors:** Kay Lucek, Irena Klečková, Charlotte J. Wright, Joana I. Meier, Mark L. Blaxter, Wellcome Sanger Institute Tree of Life Management, Samples and Laboratory team, Wellcome Sanger Institute Scientific Operations: Sequencing Operations, Wellcome Sanger Institute Tree of Life Core Informatics team, Tree of Life Core Informatics collective, Project Psyche Community

**Affiliations:** 1 University of Neuchâtel, Neuchâtel, Neuchâtel, Switzerland; 2 Biology Centre Czech Academy of Sciences, České Budějovice, South Bohemia, Czech Republic; 3 Tree of Life, Wellcome Sanger Institute, Hinxton, England, UK

**Keywords:** Erebia pharte, Blind Ringlet, genome sequence, chromosomal, Lepidoptera

## Abstract

We present a genome assembly from a female specimen of *Erebia pharte* (Blind Ringlet; Arthropoda; Insecta; Lepidoptera; Nymphalidae). The genome sequence has a total length of 616.83 megabases. Most of the assembly (98.95%) is scaffolded into 20 chromosomal pseudomolecules, including the Z sex chromosome. The mitochondrial genome has also been assembled, with a length of 15.21 kilobases. Gene annotation of this assembly by Ensembl identified 15,135 protein-coding genes. This work is part of Project Psyche, a collaborative programme generating genomes for European butterflies and moths.

## Species taxonomy

Eukaryota; Opisthokonta; Metazoa; Eumetazoa; Bilateria; Protostomia; Ecdysozoa; Panarthropoda; Arthropoda; Mandibulata; Pancrustacea; Altocrustacea; Allotriocarida; Hexapoda; Insecta; Dicondylia; Pterygota; Neoptera; Eumetabola; Endopterygota; Aparaglossata; Panorpida; Amphiesmenoptera; Lepidoptera; Glossata; Neolepidoptera; Heteroneura; Ditrysia; Obtectomera; Papilionoidea; Nymphalidae; Satyrinae; Erebiini; *Erebia*; *Erebia pharte* (Hubner, 1803-1804) (NCBI:txid111906)

## Background

*Erebia pharte*, the Blind Ringlet, is a small, brownish *Erebia* species with orange spots on the upper and lower sides of the wings. The orange spots lack central black dots, which is why the species is called the Blind Ringlet. The species may resemble individuals of *Erebia melampus*, which can also lack black centres in the ocelli ( Sonderegger, 2005). *Erebia pharte* differs from *E. manto* and *E. eriphyle* in the shape of the orange ocelli.

*Erebia pharte* is distributed in the Alps and the Slovak Carpathians (Tatra Mountains) ( Panigaj *et al.*, 2015; Tolman & Lewington, 2009). The species is also reported from Romania (see Sonderegger, 2005), but probably does not occur there ( Cuvelier & Dincă, 2007). Similarly, the species has been reported from the Vosges ( Tolman & Lewington, 2009), but it probably does not occur there either ( Arnscheid & Roos, 1981; Sonderegger, 2005). *Erebia pharte* belongs to the *Erebia epiphron* species group and is related to *Erebia epiphron*, *E. orientalis*, *E. flavofasciata* and *E. melampus* ( Augustijnen *et al.* , 2024; Klečková *et al.*, 2023).

Larvae feed on grasses and sedges (Poaceae and Cyperaceae), including *Carex ferruginea*, *C. flacca*, *Festuca ovina*, *F. quadriflora*, *F. rubra* and *Nardus stricta* ( Clarke, 2024). The habitat of the species includes mountain meadows and pastures, as well as subalpine and alpine meadows with taller grasses. The species has a two-year life cycle with four larval stages, and it overwinters in the first and third larval instars ( Sonderegger, 2005). *Erebia pharte* is currently assessed as Least Concern by the IUCN, with a stable population trend ( van Swaay *et al.* , 2025).

We present a chromosome-level, haplotype-resolved genome sequence of the Blind Ringlet, *Erebia pharte*, sequenced as part of Project Psyche ( Wright *et al.*, 2025). The sequence data were derived from a specimen (Figure 1) collected from Gadmen, Bern, Switzerland.

**Figure 1.**
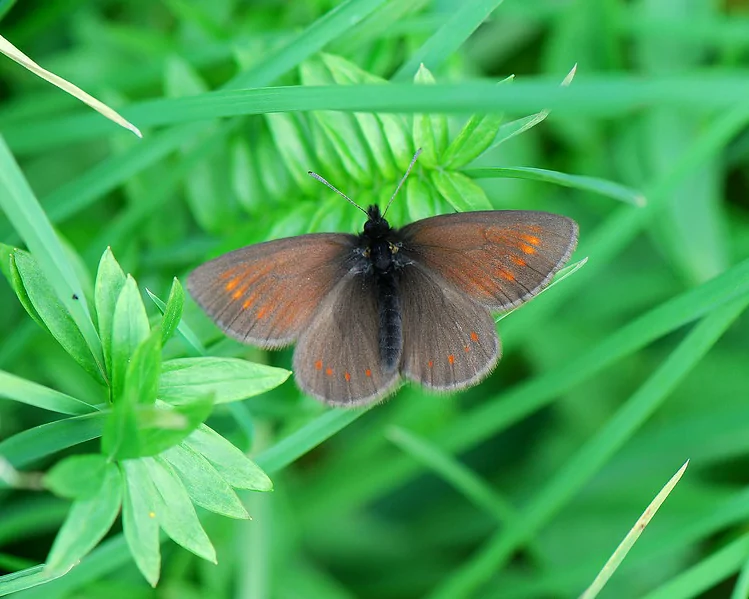
Voucher photograph of the *Erebia pharte* (ilErePhar2) specimen used for genome sequencing.

## Methods

### Sample acquisition

The specimen used for genome sequencing was an adult female *Erebia pharte* (specimen ID SAN20001732, ToLID ilErePhar2; Figure 1), collected from Gadmen, Bern, Switzerland (latitude 46.7317, longitude 8.4244; elevation 1,850 m) on 2022-07-07. The specimen was collected and identified by Kay Lucek (University of Neuchâtel).

### Nucleic acid extraction

Protocols for high molecular weight (HMW) DNA extraction developed at the Wellcome Sanger Institute (WSI) Tree of Life Core Laboratory are available on protocols.io ( Howard *et al.*, 2025). For HMW DNA extraction, tissue from the whole organism of ilErePhar2 was homogenised by powermashing using a PowerMasher II tissue disruptor. HMW DNA was extracted in the WSI Scientific Operations core using the Automated MagAttract v2 protocol. DNA was sheared into an average fragment size of 12–20 kb following the Megaruptor®3 for LI PacBio protocol. Sheared DNA was purified by manual SPRI (solid-phase reversible immobilisation). The concentration of the sheared and purified DNA was assessed using a Nanodrop spectrophotometer and Qubit Fluorometer using the Qubit dsDNA High Sensitivity Assay kit. Fragment size distribution was evaluated by running the sample on the Femto Pulse system. For this sample, the final post-shearing DNA had a Qubit concentration of 24.2 ng/μL and a yield of 1,403.60 ng, with a fragment size of 13.4 kb. The 260/280 spectrophotometric ratio was 2.01, and the 260/230 ratio was 2.47. The Genomic Quality Number (GQN) was 5.6.

### PacBio HiFi library preparation and sequencing

Library preparation and sequencing took place in the WSI Scientific Operations core.

Library 6771STDY14439721 was prepared with the SMRTbell Prep Kit 3.0 (Pacific Biosciences) according to the manufacturer’s instructions.

The kit includes reagents for end repair/A-tailing, adapter ligation, post-ligation SMRTbell bead clean-up, and nuclease treatment. Size selection and clean-up were performed using diluted AMPure PB beads (Pacific Biosciences). DNA concentration was quantified with a Qubit Fluorometer v4.0 (Thermo Fisher Scientific) and the Qubit 1X dsDNA HS assay kit. Final library fragment size was assessed with the Agilent Femto Pulse Automated Pulsed Field CE Instrument (Agilent Technologies) using the gDNA 55 kb BAC analysis kit.

Library 6771STDY14439721 was sequenced on a Revio instrument (Pacific Biosciences, California, USA). The prepared libraries selected for multiplexing were pooled based on genome size, library molarity and the intended plex level. Primers were annealed and polymerases were bound to generate circularised complexes following the manufacturer’s instructions. Complexes were purified using SMRTbell beads, diluted to the Revio loading concentration, and spiked with a Revio sequencing internal control. The pooled libraries were sequenced on a Revio 25M Plex SMRT cell in a 4-plex run. SMRT Link software (Pacific Biosciences) was used to configure and monitor the run and to perform primary and secondary data analysis.

### Hi-C

***Sample preparation and crosslinking***

Hi-C data were generated from the ilErePhar2 whole organism sample using the Arima-HiC v2 kit (Arima Genomics). Following the manufacturer’s instructions, tissue was fixed and DNA crosslinked using TC buffer to a final formaldehyde concentration of 2%. The tissue was homogenised using the Diagnocine Power Masher-II. Crosslinked DNA was digested with a restriction enzyme master mix, biotinylated, and ligated. Clean-up was performed with SPRISelect beads before library preparation. DNA concentration was measured with the Qubit Fluorometer (Thermo Fisher Scientific) and Qubit HS Assay Kit. The biotinylation percentage was estimated using the Arima-HiC v2 QC beads.

***Hi-C library preparation and sequencing***

Biotinylated DNA constructs were fragmented using a Covaris E220 sonicator and size-selected to 400–600 bp using SPRISelect beads. DNA was enriched with Arima-HiC v2 kit Enrichment beads. End repair, A-tailing, and adapter ligation were carried out with the NEBNext Ultra II DNA Library Prep Kit (New England Biolabs), following a modified protocol where library preparation occurs while DNA remains bound to the Enrichment beads. Libraries were amplified with KAPA HiFi HotStart mix and a custom Unique Dual Index (UDI) barcode set (Integrated DNA Technologies). Depending on sample concentration and biotinylation percentage determined at the crosslinking stage, libraries were amplified with 10–16 PCR cycles. Post-PCR clean-up was performed with SPRISelect beads. Libraries were quantified using the AccuClear Ultra High Sensitivity dsDNA Standards Assay Kit (Biotium) and a FLUOstar Omega plate reader (BMG Labtech).

Prior to sequencing, libraries were normalised to 10 ng/μL. Normalised libraries were quantified again to create equimolar and/or weighted 2.8 nM pools. Pool concentrations were checked using the Agilent 4200 TapeStation (Agilent) with High Sensitivity D500 reagents before sequencing. Libraries were sequenced on the Illumina NovaSeq X, generating paired-end 150-bp reads.

### Genome assembly

Prior to assembly of the PacBio HiFi reads, a database of *k*-mer counts ( *k* = 31) was generated from the filtered reads using FastK. The *k*-mer frequency distribution was analysed with GenomeScope (version 2.1.0) ( Ranallo-Benavidez *et al.*, 2020), providing fitted point estimates of genome size, heterozygosity and repeat content.

The HiFi reads were assembled using Hifiasm ( Cheng *et al.*, 2021) with the --primary option. Haplotypic duplications were identified and removed using purge_dups ( Guan *et al.*, 2020). The Hi-C reads ( Rao *et al.* , 2014) were mapped to contigs using bwa-mem2 ( Vasimuddin *et al.*, 2019), and the contigs were scaffolded in YaHS ( Zhou *et al.*, 2023) with the --break option for handling potential misassemblies. The scaffolded assemblies were evaluated using Gfastats ( Formenti *et al.* , 2022), BUSCO ( Manni *et al.*, 2021) and MerquryFK ( Rhie *et al.*, 2020).

The mitochondrial genome was assembled using MitoHiFi ( Uliano-Silva *et al.* , 2023), which runs MitoFinder ( Allio *et al.*, 2020) and uses these annotations to select the final mitochondrial contig and to ensure the general quality of the sequence, using the mitochondrial genome of *Minois paupera* (NC_085606.1) as the reference.

### Assembly curation

The assembly was decontaminated using the Assembly Screen for Cobionts and Contaminants ( ASCC) pipeline. The flat files and maps for curation were generated with TreeVal. Manual curation was conducted primarily in PretextView and HiGlass ( Kerpedjiev *et al.* , 2018). Scaffolds were visually inspected and corrected as described by Howe *et al*. (2021). Manual corrections included 21 breaks and 30 joins, which reduced the scaffold count by 16.5%. The curation process is described at sanger-tol/curation-resources. A Hi-C contact map of the primary assembly was generated with PretextSnapshot.

### Assembly quality assessment

The MerquryFK tool ( Rhie *et al.*, 2020), run in a Singularity container ( Kurtzer *et al.*, 2017), was used to evaluate *k*-mer completeness and assembly quality for the primary and alternate haplotypes using the *k*-mer databases ( *k* = 31) computed prior to genome assembly. The analysis outputs included assembly QV scores and completeness statistics.

The primary assembly was assessed using BlobToolKit ( Challis *et al.*, 2020). PacBio reads were mapped to the primary assembly to derive sequence coverage, and BUSCO ( Manni *et al.*, 2021) was used to assess gene-set completeness. DIAMOND BLASTp searches of extracted BUSCO proteins and DIAMOND BLASTx searches of assembly sequences against UniProt Reference Proteomes ( Buchfink *et al.*, 2021), followed by BLASTn searches of sequences without DIAMOND BLASTx hits against the NCBI nt database ( Altschul *et al.*, 1990), supported taxonomic assignment. Coverage, sequence composition, taxonomic assignment and BUSCO results were integrated into a BlobDir for interactive inspection and visualisation. The snail and blob plots were rendered locally from this hosted BlobDir using BlobTk v0.8.3. The scaffold-length radial scale function for the snail plot was set to linear ( Challis & Blaxter, 2026). The blob plot used circular markers with area proportional to scaffold length ( Challis *et al.*, 2020).

We used *busco-alg-painter* v0.1.0, a Python reimplementation and extension of *lep_busco_painter*, to paint Merian elements along chromosomes. Merian elements represent the 32 ancestral linkage groups reconstructed for Lepidoptera ( Wright *et al.*, 2024). The BUSCO-to-Merian reference assignments were derived from *lep_busco_painter*. Complete single-copy and duplicated BUSCO genes from the lepidoptera_odb10 results were assigned to Merian elements. Chromosome identifiers and lengths were obtained from NCBI Datasets, and BUSCO positions were plotted on chromosomes drawn to scale.

## Genome sequence report

### Sequence data

PacBio sequencing of the *E. pharte* specimen generated 21.27 Gb (gigabases) from 2.48 million reads. We assembled the genome from these reads. GenomeScope analysis using a *p* = 2 model gave a *k*-mer-based haploid genome size estimate of 594.20 Mb, with heterozygosity of 0.84% and repeat content of 44.11%; the full-model fit was 98.55% (Figure 2). The GenomeScope estimates guided expectations for the assembly. The sequencing data provided approximately 35× coverage of the primary assembly. Hi-C sequencing produced 91.16 Gb from 301.87 million reads, which were used to scaffold the primary assembly. Table 1 summarises the specimen and sequencing details.

**Figure 2.**
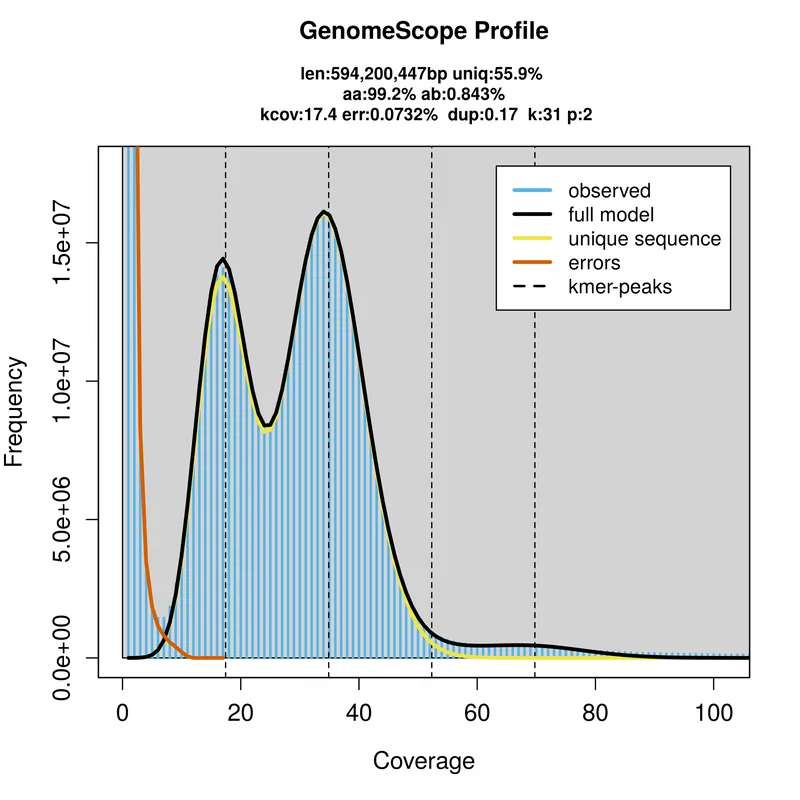
GenomeScope model fitted to the *k*-mer frequency distribution. The plot shows the observed and modelled *k*-mer spectra used to estimate genome size, heterozygosity and repeat content from unassembled sequencing reads.

**Table 1.** Specimen and sequencing data for *E. pharte* (BioProject PRJEB75866)

**Platform**	**PacBio HiFi**	**Hi-C**
**ToLID**	ilErePhar2	ilErePhar2
**Specimen ID**	SAN20001732	SAN20001732
**BioSample (source individual)**	SAMEA114286227	SAMEA114286227
**BioSample (tissue)**	SAMEA114286288	SAMEA114286288
**Tissue**	whole organism	whole organism
**Instrument**	Revio (1 run)	Illumina NovaSeq X
**Library ID & run accession(s)**	6771STDY14439721: ERR13158709	ERR13148270
**Read count total**	2.48 million reads	301.87 million read pairs
**Base count total**	21.27 Gb	91.16 Gb

### Assembly statistics

The primary assembly was generated and an alternate haplotype assembly was also deposited at scaffold level in INSDC databases. The primary assembly is proposed as the reference assembly for this species. It has a total length of 616.83 Mb in 70 scaffolds (excluding organelle sequences), with 134 gaps, and a scaffold N50 of 31.3 Mb (Table 2). The primary assembly had a scaffold auN of 34.0 Mb.

**Table 2.** Genome assembly statistics for *E. pharte*

**Genome assembly**	**Primary assembly**	**Alternate haplotype assembly**
**Assembly name**	ilErePhar2.1	ilErePhar2.1 alternate haplotype
**Assembly accession**	GCA_965112705.1	GCA_965112695.1
**Assembly level**	chromosome	scaffold
**Span (Mb)**	616.83	582.07
**Number of chromosomes**	20	-
**Number of contigs**	204	3,036
**Contig N50 (Mb)**	8.12	0.64
**Number of scaffolds (excluding organelle sequences)**	70	3,008
**Scaffold N50 (Mb)**	31.3	0.65
**Scaffold auN (Mb)**	34.0	-
**Longest scaffold length (Mb)**	52.41	3.66
**Sex chromosomes**	Z	-
**Organelles**	Mitochondrial genome: 15.21 kb	-

Most of the primary assembly sequence (98.95%) was assigned to 20 chromosome-level scaffolds, representing 19 autosomes and the Z sex chromosome. These chromosome-level scaffolds, confirmed by Hi-C data, are named according to size (Figure 3; Table 3). Chromosome painting of the primary assembly with Merian elements illustrates the distribution of orthologues along chromosomes and highlights patterns of chromosomal evolution relative to Lepidopteran ancestral linkage groups (Figure 4). The Z chromosome of the primary assembly was assigned based on the presence of the ancestral Merian element MZ ( Wright *et al.*, 2024).

The mitochondrial genome was also assembled (length 15.21 kb, OZ218833.1). This sequence is included as a contig in the multifasta file of the primary genome submission and as a standalone record.

**Figure 3.**
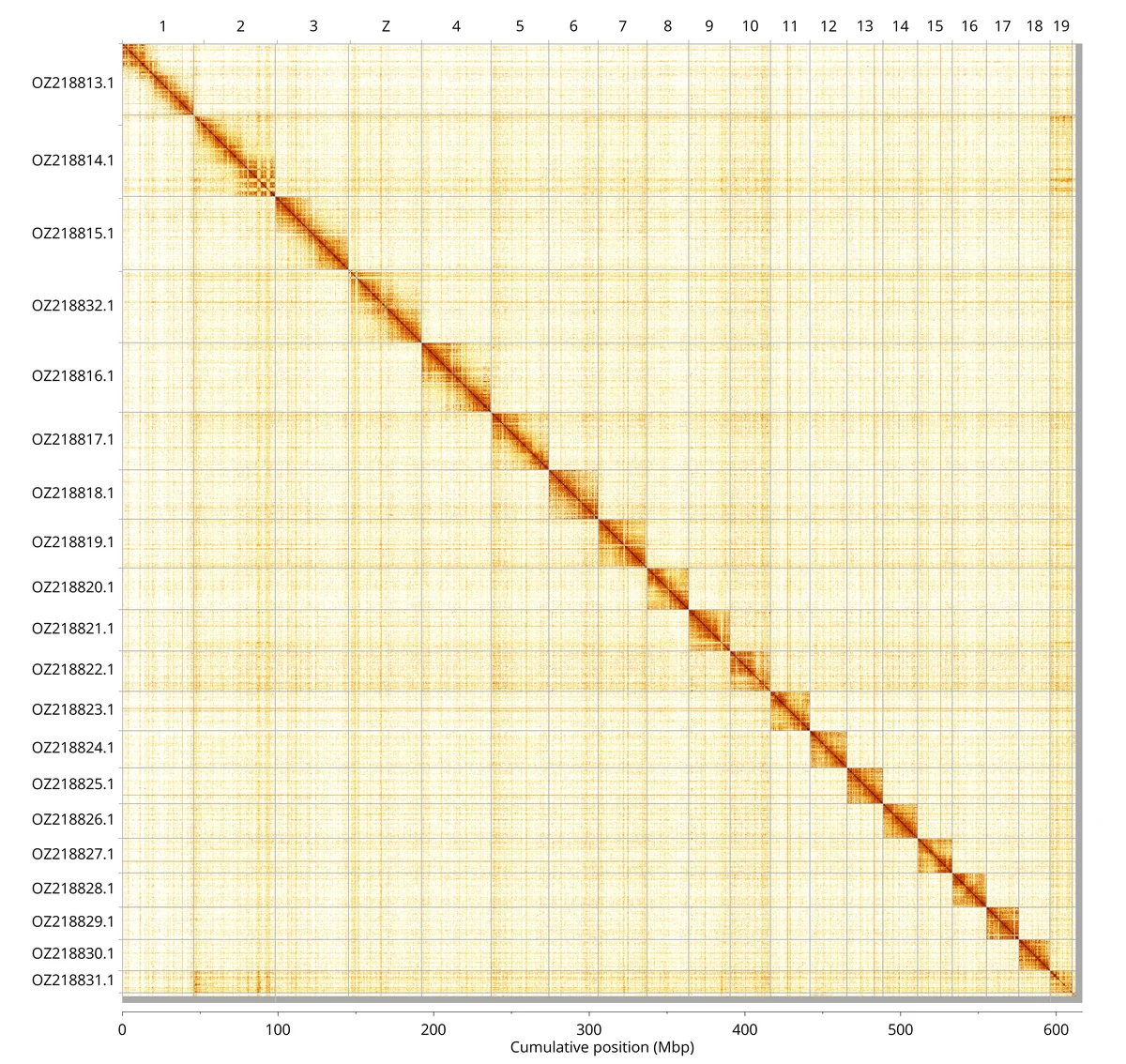
Hi-C contact map of the *E. pharte* primary assembly. Assembled chromosomes are shown in order of size and labelled along the axes. The plot was generated using PretextSnapshot.

**Table 3.** Chromosomal pseudomolecules in the primary genome assembly of *E. pharte* ilErePhar2

**INSDC accession**	**Molecule**	**Length (Mb)**	**GC%**	**Assigned Merian elements**
OZ218813.1	1	52.49	37.50	M2; M23; M30
OZ218814.1	2	47.12	37.50	M14; M16; M29
OZ218815.1	3	46.90	37.50	M10; M12
OZ218816.1	4	44.69	37.50	M21; M8
OZ218817.1	5	36.96	37.50	M17; M20; M25
OZ218818.1	6	31.79	37.50	M22; M27
OZ218819.1	7	31.30	37.50	M13; M28
OZ218820.1	8	26.80	37.50	M1
OZ218821.1	9	26.54	37.50	M19; M26
OZ218822.1	10	26.04	37.50	M24; M31
OZ218823.1	11	25.31	37.50	M3
OZ218824.1	12	23.82	37.50	M5
OZ218825.1	13	23.07	37	M9
OZ218826.1	14	22.28	37.50	M7
OZ218827.1	15	22.24	37.50	M4
OZ218828.1	16	21.95	37.50	M6
OZ218829.1	17	20.89	38	M11
OZ218830.1	18	20.04	37.50	M15
OZ218831.1	19	14.23	38	M30
OZ218832.1	Z	45.88	37	M18; MZ

**Figure 4.**
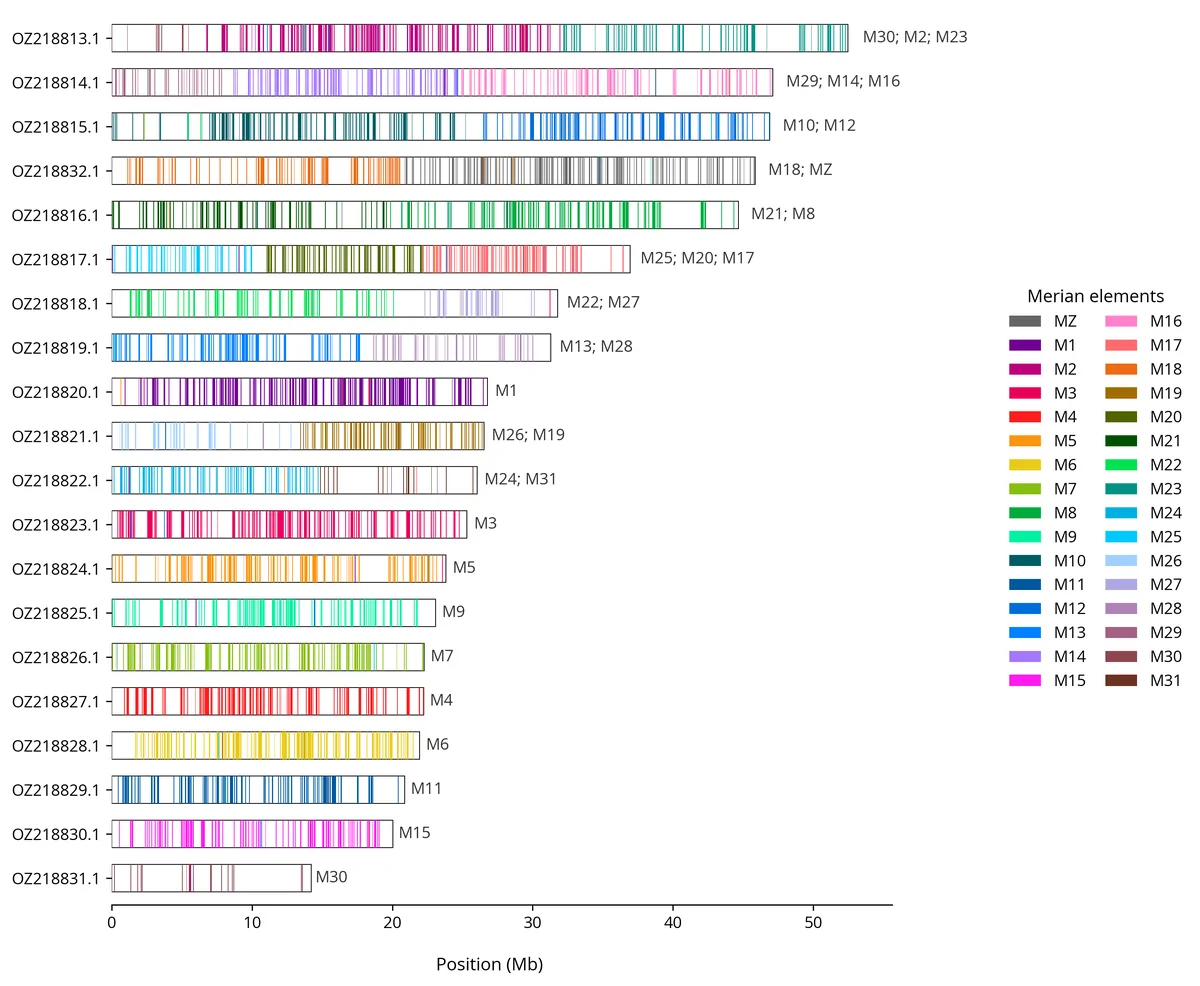
Merian elements painted across chromosomes in the ilErePhar2.1 primary assembly of *E. pharte*. Chromosomes are drawn to scale, with the positions of orthologues shown as coloured bars. Each orthologue is coloured by the Merian element that it belongs to. All orthologues which could be assigned to Merian elements are shown.

### Assembly quality metrics

The combined primary and alternate assemblies achieve an estimated QV of 63.0. The *k*-mer completeness is 85.24% for the primary assembly, 75.25% for the alternate haplotype, and 98.78% for the combined assemblies (Figure 5).

**Figure 5.**
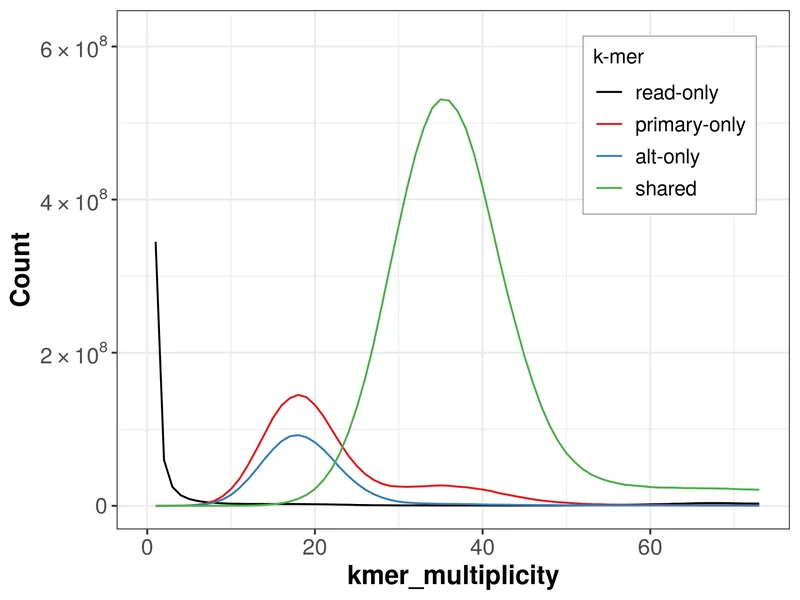
Evaluation of *k*-mer completeness using MerquryFK. This plot illustrates the recovery of *k*‐mers from the original read data in the primary assembly, the alternate haplotype assembly and the combined assemblies. The horizontal axis represents *k*‐mer multiplicity, and the vertical axis shows the number of *k*‐mers. The black curve represents *k*‐mers that appear in the reads but are not assembled. The green curve corresponds to *k*‐mers shared by both haplotypes, and the red and blue curves show *k*‐mers found only in one of the haplotypes.

BUSCO v.5.7.1 analysis using the lepidoptera_odb10 reference set ( *n* = 5,286) ( Kriventseva *et al.*, 2019) identified 98.5% of the expected gene set (single = 97.6%, duplicated = 0.9%).

The snail plot in Figure 6 provides a summary of assembly contiguity, cumulative scaffold count, sequence composition and BUSCO completeness for the primary assembly ( Challis & Blaxter, 2026). The relative sizes of the longest scaffold, scaffold N50 and N90, together with the dark-grey scaffold-length distribution, show how much of the assembly is contained in long scaffolds. The outer composition track shows variation in GC, AT and N content, while the central track shows the cumulative scaffold count and the BUSCO inset summarises gene-set completeness.

The blob plot in Figure 7 displays scaffolds from the primary assembly by GC proportion and base coverage. Marker area is proportional to scaffold length, and colour indicates taxonomic assignment ( Challis *et al.*, 2020).

**Figure 6.**
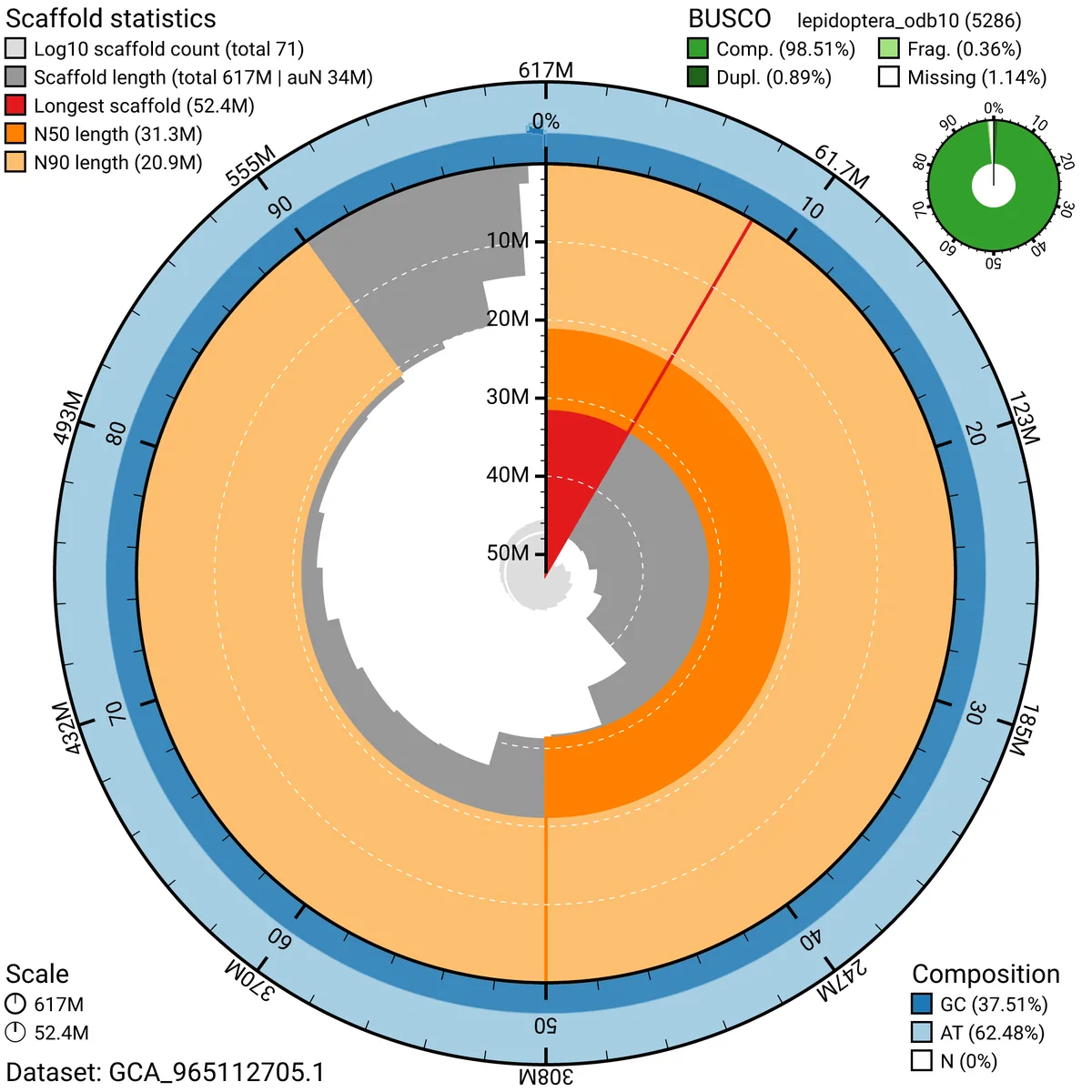
Assembly metrics for ilErePhar2.1, the primary assembly. The BlobToolKit snail plot provides an overview of assembly metrics and BUSCO gene completeness. The circumference represents the length of the primary assembly, and the main plot is divided into 1,000 bins around the circumference. The outermost blue tracks display the distribution of GC, AT, and N percentages across the bins. Scaffolds are arranged clockwise from longest to shortest and are depicted in dark grey. Scaffold length is shown on a linear radial scale, scaled to the longest scaffold. Red shading indicates the longest scaffold, and the deeper orange and pale orange shading represent the N50 and N90 lengths. The summary statistics include scaffold auN, the area under the scaffold Nx curve. The light grey spiral at the centre shows the cumulative scaffold count on a logarithmic scale. A summary of complete, fragmented, duplicated, and missing BUSCO genes in the lepidoptera_odb10 set is presented at the top right. The assembly can be explored interactively in the BlobToolKit viewer.

**Figure 7.**
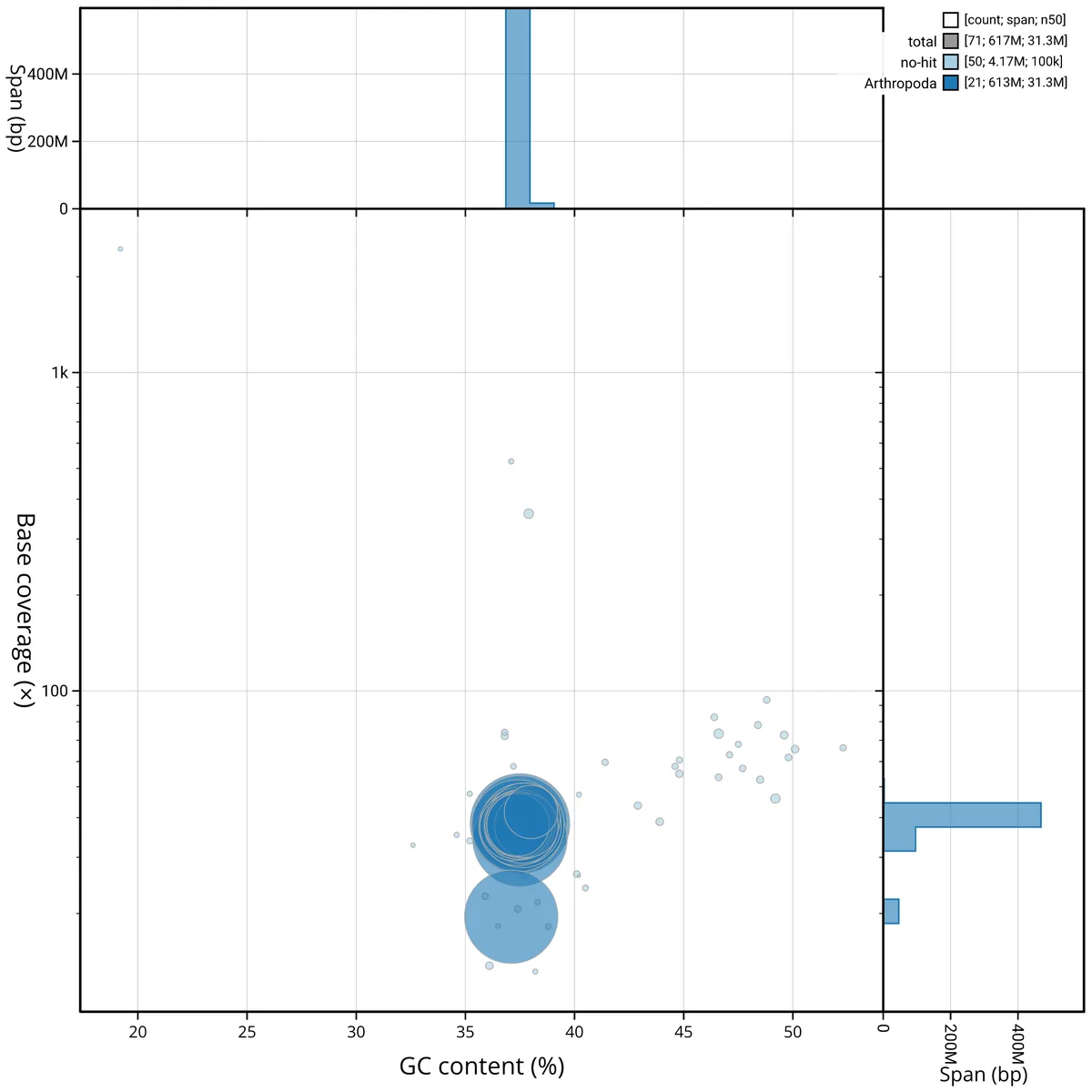
BlobToolKit blob plot for ilErePhar2.1. The plot shows base coverage (vertical axis) and GC content (horizontal axis). Circles represent scaffolds; marker area is proportional to scaffold length, and colour indicates taxonomic assignment. The histograms along the axes display the total length of sequences distributed across different levels of coverage and GC content. The assembly can be explored interactively in the BlobToolKit viewer.

Table 4 lists the assembly metric benchmarks adapted from Rhie *et al.*. (2021) the Earth BioGenome Project (EBP) Report on Assembly Standards. The three-part EBP metric, calculated for the primary assembly, is **6.C.Q64**.

**Table 4.** Earth BioGenome Project summary metrics for the *E. pharte* assembly

**Measure**	**Value**	***Benchmark***
EBP summary (primary)	6.C.Q64	*6.C.Q40*
Contig N50 length	8.12 Mb	*> 1 Mb*
Scaffold N50 length	31.30 Mb	*= chromosome N50*
Consensus quality (QV)	Primary: 64.2; alternate: 62.5; combined: 63.0	*≥ 40*
*k*-mer completeness	Primary: 85.24%; alternate: 75.25%; combined: 98.78%	*> 90%*
BUSCO	C:98.5% [S:97.6%, D:0.9%], F:0.4%, M:1.1%, n:5,286	*S > 90%; D < 5%*
Percentage of assembly assigned to chromosomes	98.95%	*≥ 90%*

**Notes:** The EBP summary uses log10(Contig N50); chromosome-level (C) or log10(Scaffold N50); Q (Merqury QV). BUSCO v5.7.1 using the lepidoptera_odb10 lineage dataset: C=complete; S=single-copy; D=duplicated; F=fragmented; M=missing; n=orthologues.

### Genome annotation report

The *E. pharte* genome assembly (GCA_965112705.1) was annotated by Ensembl at the European Bioinformatics Institute (EBI). The annotation comprises 33,980 transcripts from 15,135 protein-coding genes and 6,348 non-coding genes. The average transcript length is 17,381.29 bp, with an average of 1.58 coding transcripts per gene and 6.51 exons per transcript. For further information, please refer to the Ensembl annotation page.

### Data availability

European Nucleotide Archive: Erebia pharte (blind ringlet). Accession number: PRJEB75866; URL: https://identifiers.org/ena.embl/PRJEB75866. The genome sequence is released openly for reuse. The *E. pharte* genome sequencing initiative is part of the Sanger Institute Tree of Life Programme ( PRJEB43745) and Project Psyche ( PRJEB71705). All raw sequence data and the primary and alternate assemblies have been deposited in INSDC databases. Raw data and assembly accession identifiers are reported in Table 1 and Table 2.

Pipelines used for genome assembly at the WSI Tree of Life are available at https://pipelines.tol.sanger.ac.uk/pipelines. Table 5 lists software versions used in this study.

**Table 5.** Software versions and sources used for *E. pharte*

**Software**	**Version**	**Source**
BLAST	2.14.0	https://ftp.ncbi.nlm.nih.gov/blast/executables/blast+/
BlobToolKit	4.4.5	https://github.com/genomehubs/blobtoolkit
BlobTk (hosted BlobDir)	0.5.1	https://github.com/genomehubs/blobtk
BlobTk (snail and blob plot renderer)	0.8.3	https://github.com/genomehubs/blobtk
BUSCO	5.7.1	https://gitlab.com/ezlab/busco
busco-alg-painter	0.1.0	https://github.com/Karenvn/busco-alg-painter
bwa-mem2	2.2.1	https://github.com/bwa-mem2/bwa-mem2
DIAMOND	2.1.8	https://github.com/bbuchfink/diamond
fasta_windows	0.2.4	https://github.com/tolkit/fasta_windows
FastK	1.2	https://github.com/thegenemyers/FASTK
GenomeScope	2.1.0	https://github.com/tbenavi1/genomescope2.0
Gfastats	1.3.6	https://github.com/vgl-hub/gfastats
Hifiasm	0.19.8-r603	https://github.com/chhylp123/hifiasm
HiGlass	1.13.4	https://github.com/higlass/higlass
MerquryFK	1.1.0-c1	https://github.com/thegenemyers/MERQURY.FK
Minimap2	2.28-r1209	https://github.com/lh3/minimap2
MitoHiFi	3.2.2	https://github.com/marcelauliano/MitoHiFi
MultiQC	1.14; 1.17 and 1.18	https://github.com/MultiQC/MultiQC
Nextflow	24.10.4	https://github.com/nextflow-io/nextflow
PretextSnapshot	0.0.6	https://github.com/sanger-tol/PretextSnapshot
PretextView	1.0.3	https://github.com/sanger-tol/PretextView
purge_dups	1.2.3	https://github.com/dfguan/purge_dups
samtools	1.21	https://github.com/samtools/samtools
sanger-tol/ascc	0.1.0	https://github.com/sanger-tol/ascc
sanger-tol/blobtoolkit	v0.7.1	https://github.com/sanger-tol/blobtoolkit
sanger-tol/curationpretext	1.4.2	https://github.com/sanger-tol/curationpretext
Seqtk	1.4-r122	https://github.com/lh3/seqtk
Singularity	3.9.0	https://github.com/sylabs/singularity
TreeVal	1.1.0	https://github.com/sanger-tol/treeval
YaHS	1.1a.2	https://github.com/c-zhou/yahs

### Author information

Contributors are listed at the following links:

- Members of the Wellcome Sanger Institute Tree of Life Management, Samples and Laboratory team
- Members of Wellcome Sanger Institute Scientific Operations – Sequencing Operations
- Members of the Wellcome Sanger Institute Tree of Life Core Informatics team
- Members of the Tree of Life Core Informatics collective
- Members of the Project Psyche Community

### Wellcome Sanger Institute – Legal and Governance

The materials that have contributed to this genome note have been supplied by a Tree of Life collaborator. The Wellcome Sanger Institute employs a process whereby due diligence is carried out proportionate to the nature of the materials themselves, and the circumstances under which they have been/are to be collected and provided for use. The purpose of this is to address and mitigate any potential legal and/or ethical implications of receipt and use of the materials as part of the research project, and to ensure that in doing so, we align with best practice wherever possible. The overarching areas of consideration are:

- Ethical review of provenance and sourcing of the material
- Legality of collection, transfer and use (national and international).

Each transfer of samples is undertaken according to a Research Collaboration Agreement or Material Transfer Agreement entered into by the Tree of Life collaborator, Genome Research Limited (operating as the Wellcome Sanger Institute), and in some circumstances, other Tree of Life collaborators.
