# Supplementary material for: The genome sequence of the Blind Ringlet, Erebia pharte (Hubner, 1803-1804) (Lepidoptera: Nymphalidae)
Source: Wellcome Open Res. 2026 Sep 4;11:592. [Version 1] doi: 10.12688/wellcomeopenres.27705.1 (PMC13673080; doi:10.12688/wellcomeopenres.27705.1)
Supplement: Peer review history

# Reviewer report: Reviewer response for version 1

**DOI:** 10.21956/wellcomeopenres.30496.r169050

**Published:** 2026-10-15

**Authors:** Ricardo Rodriguez-de-la-vega

**Affiliations:** 1 Université Paris-Saclay, Gif-sur-Yvette, France

Comments on Lucek et al., "The genome sequence of the Blind Ringlet..."

 In this Psyche project Data Note, the authors present an Earth BioGenome Project (EBP) reference-level, haplotype-resolved assembly of a female specimen of E *rebia pharte*, a nymphalid moth commonly known as the blind ringlet. Unlike most other Psyche project assemblies, the alternative haplotype produced is highly fragmented (>3,000 scaffolds, EBP metric: 5.5.62).

 Nucleic acid extraction, library preparation (HiFi and Hi-C), and sequencing were carried out at the Wellcome Sanger Institute (WSI) Scientific Operations core or followed protocols developed by the WSI Tree of Life Core Laboratory. The assemblies were generated using hifiasm on HiFi reads scaffolded with Hi-C reads. The primary assembly was manually curated in PretextView and HiGlass using Hi-C reads mapped onto the assembly. As is now common for Psyche project Data Notes, the supporting materials include tables for:

- "Specimen and sequencing data",
- "Genome assembly statistics",
- "Chromosomal pseudomolecules in the primary assembly",
- EBP "summary statistics",
- "Software versions and sources".

Supporting figures include:

- GenomeScope2 FastK 31-mer frequency distribution,
- PretextSnapshot of Hi-C contacts,
- Merian elements on length-scaled chromosome pseudomolecules of the primary assembly,
- MerquryFK read-only, haplotype-specific, and combined assemblies 31-mer distributions,
- Snail and blob plots of the primary assembly scaffolds.

I detected no departures from the well-established protocols associated with Psyche project genome assemblies. Beyond the incremental nature of the resources presented here (as of September 5th, this is the twenty-second *Erebia* reference-level assembly released by the Psyche or Darwin Tree of Life projects in Wellcome Open Research, and the 16th obtained from a female specimen in the genus), their most interesting contribution will emerge once researchers begin analyzing the data in a comparative genomics framework. For instance, among the *Erebia* assemblies released by DToL or Psyche projects, W sex chromosomes are conspicuously absent from several female assemblies (e.g., WOR_11-650; WOR_11-588), while in others, the W chromosome is the largest (WOR_11-589; WOR_10-425) or the smallest assembled chromosome (WOR_11-75).

 For convenience, I am including links to the DToL quality check and Genomes on a Tree page for this specimen/species.

 https://tolqc.cog.sanger.ac.uk/tol/insects/Erebia_pharte/

 https://goat.genomehubs.org/record?recordId=111906&result=taxon&taxonomy=ncbi#Erebia%20pharte

Are the datasets clearly presented in a usable and accessible format, and the assembly and annotation available in an appropriate subject-specific repository?

Yes

Are sufficient details of the sequencing and extraction, software used, and materials provided to allow replication by others?

Yes

Are the rationale for sequencing the genome and the species significance clearly described?

Yes

Are the protocols appropriate and is the work technically sound?

Yes

Reviewer Expertise:

Evolutionary Genomics; Bioinformatics

I confirm that I have read this submission and believe that I have an appropriate level of expertise to confirm that it is of an acceptable scientific standard.

## References

[1] : The genome sequence of the Sudeten Ringlet, Erebia sudetica Dalman, 1816 (Lepidoptera: Nymphalidae). *Wellcome Open Research* .2026;11: 10.12688/wellcomeopenres.25830.1 10.12688/wellcomeopenres.25830.1

[2] : The genome sequence of the de Prunner’s Ringlet, Erebia triaria von Prunner, 1798 (Lepidoptera: Nymphalidae). *Wellcome Open Research* .2026;10: 10.12688/wellcomeopenres.24693.2 10.12688/wellcomeopenres.24693.2 PMC1242362140949818

# Reviewer report: Reviewer response for version 1

**Published:** 2026-09-04

**Authors:** Automated Benchmarking

**Affiliations:** 1 Wellcome Sanger Institute, Hinxton, UK

As this article is a Genome Note, Automated Benchmarking has been used to determine whether the reported assembly passes three defined checks established by the Earth BioGenome Project: the raw and assembled data are publicly available, the Earth BioGenome Project three-part metric is met, and the BUSCO score passes the stated threshold. For Erebia pharte (ilErePhar2), all three checks passed. Data available: pass. ENA study PRJEB75866, raw-read accessions ERR13158709 and ERR13148270, and assembly accessions GCA_965112705.1 and GCA_965112695.1 are public. Earth BioGenome Project three-part metric: pass. The primary assembly is reported as 6.C.Q64, meeting the 6.C.Q40 reference standard. BUSCO: pass. The primary assembly, which is proposed as the reference assembly for this species, has 98.5% complete BUSCOs (97.6% single-copy and 0.9% duplicated). It meets the target of greater than 90% complete and single-copy BUSCOs. This decision uses values reported in the Genome Note and public accession records.

Reviewer Expertise:

NA

I confirm that I have read this submission and believe that I have an appropriate level of expertise to confirm that it is of an acceptable scientific standard.
